# The main but not the accessory olfactory system is involved in the processing of socially relevant chemosignals in ungulates

**DOI:** 10.3389/fnana.2012.00039

**Published:** 2012-09-19

**Authors:** Matthieu Keller, Frédéric Lévy

**Affiliations:** ^1^INRA, UMR 85 Physiologie de la Reproduction et des ComportementsNouzilly, France; ^2^CNRS, UMR 7247 Physiologie de la Reproduction et des ComportementsNouzilly, France; ^3^Université François Rabelais de ToursTours, France

**Keywords:** maternal behavior, sociosexual interactions, olfactory systems, male effect, olfactory learning, vomeronasal organ

## Abstract

Ungulates like sheep and goats have, like many other mammalian species, two complementary olfactory systems. The relative role played by these two systems has long been of interest regarding the sensory control of social behavior. The study of ungulate social behavior could represent a complimentary alternative to rodent studies because they live in a more natural environment and their social behaviors depend heavily on olfaction. In addition, the relative size of the main olfactory bulb (MOB) [in comparison to the accessory olfactory bulb (AOB)] is more developed than in many other lissencephalic species like rodents. In this review, we present data showing a clear involvement of the main olfactory system in two well-characterized social situations under olfactory control in ungulates, namely maternal behavior and offspring recognition at birth and the reactivation of the gonadotropic axis of females exposed to males during the anestrous season. In conclusion, we discuss the apparent discrepancy between the absence of evidence for a role of the vomeronasal system in ungulate social behavior and the existence of a developed accessory olfactory system in these species.

## Introduction

The sense of smell is of primary importance for social recognition among mammals and is mediated by the main and the accessory olfactory systems. These olfactory systems differ both in their organization and in their function. The main olfactory system is involved in the processing of volatile odors detected at the level of the main olfactory epithelium in the nasal cavity. Sensory neurons send axons to glomerular cell layer of the main olfactory bulb (MOB) where they synapse with dendrites of mitral and tufted cells. The olfactory information is then conveyed to several primary olfactory structures including the anterior olfactory nucleus, the olfactory tubercle, the piriform cortex, the posterolateral cortical amygdala, or the entorhinal cortex (Petrulis, 2009). By contrast, the accessory olfactory system is involved in the detection of non-volatile odors through receptors localized in the epithelium of the vomeronasal organ (VNO). These receptors synapse onto mitral cells of the accessory olfactory bulb (AOB) which then project mainly to the medial amygdala (Halpern and Martínez-Marcos, 2003). The olfactory information then reaches the hypothalamus at the level of various structures including the medial preoptic area or the bed nucleus of the stria terminalis (Scalia and Winans, 1975).

Although the processing of olfactory information between both systems is largely independent, many levels of convergence are found between them, for example, directly at the level of the olfactory bulb (Larriva-Sahd, 2008) or more downstream at the level of the amygdala (Licht and Meredith, 1987; Kang et al., 2009). Therefore, a recurrent problem in olfactory functional studies is to elucidate the role played by each olfactory system, as well as their interactions, in the detection of olfactory information and in the regulation of social behavior (Keller et al., 2009). For example in mice, the main olfactory system can respond to 2-heptanone, a molecule known to participate to mouse social communication, but this chemosignal also activates the accessory olfactory system (Xu et al., 2005). Conversely, volatile pheromones like farnesene or dimethylpyrazine, known to be detected by the main olfactory system can also trigger cellular activation in the mice vomeronasal epithelium (Leinders-Zufall et al., 2000).

Most of our current knowledge on the involvement of the main and the accessory olfactory systems in the regulation of behavior comes from studies using very few species, mostly mice and rats living in artificial laboratory environment. However, given the wide variety of organization of both olfactory systems (Mesiami and Bhatnagar, 1998), it is likely that the respective roles played by both olfactory systems vary across mammalian species. Studying the olfactory control of social behavior in ungulates (especially sheep and goat) could represent a complimentary alternative to rodent studies because ungulates, even farm animals like sheep, live in a more natural environment and their social behaviors depend heavily on olfaction (Gelez and Fabre-Nys, 2004; Lévy et al., 2004). In addition, ungulates constitute an additional interesting model because the relative size of the MOB (in comparison to the AOB) is also more developed than in many other lissencephalic species like rodents.

In this review, we highlight the role of both olfactory systems in two well-studied social situations in sheep and goats, maternal behavior and sexual behavior (the so called “male effect”) and we then discuss the general involvement of both systems in the regulation of ungulates social behavior and in comparison to rodents.

## Olfactory systems involved in maternal behavior

In sheep, the establishment of maternal behavior is under the major influence of amniotic fluids which cover the lamb at birth. An important shift toward amniotic fluid is observed across pregnancy and parturition: while repelled by amniotic fluid throughout pregnancy, ewes become highly attracted to amniotic fluid around parturition (Lévy et al., 1983). This attraction mediates the attraction to the newborn because parturient ewes are more attracted to a model lamb smeared with amniotic fluid than the same model without amniotic fluid (Vince et al., 1985). In addition, when newborn lambs are washed to eliminate amniotic fluids at birth, maternal behavior is often disrupted, especially in inexperienced females (Lévy and Poindron, 1987). This rapid shift toward amniotic fluid is mediated by chemosensory stimuli. Indeed, when testing females in a context where the attraction/repulsion to amniotic fluid was not dependent on the presence of the lamb (test of food choice between two troughs containing food where one troughs where one trough was contaminated with amniotic fluid), it has been shown that females receiving an injection of zinc sulfate in the main olfactory cavity which destroys the main olfactory epithelium are neither repelled by nor clearly attracted to amniotic fluid (Lévy et al., 1983). By contrast, vomeronasal nerve section does not affect these attraction–repulsion responses to amniotic fluid (Lévy et al., 1995).

In sheep, the respective roles of the main and accessory olfactory systems to the display of maternal behavior were investigated in primiparous females. Anosmic mothers spent less time licking their neonates and emitted fewer maternal bleats (Lévy et al., 1995). On the other hand, mothers with only lesions of the VNO showed little disturbance of maternal care, thus underlying the importance of the main olfactory system in the establishment of maternal behavior at parturition.

In addition to the strict expression of maternal care, the establishment of a selective bond within the first few hours after parturition represents one of the essential characteristics of maternal behavior in precocial species such as ungulates. Numerous studies consistently indicate that olfaction plays a primary role in ewes' selective acceptance of lambs for nursing (Poindron and Le Neindre, 1980; Poindron et al., 1993). When females were rendered hyposmic through the use of zinc sulfate to destroy the olfactory epithelium, (Poindron, 1976; Romeyer et al., 1994; Lévy et al., 1995) or by sectioning the olfactory nerves (Morgan et al., 1975), females show no selective preference for their own young and nursed alien young indiscriminately. Similar results have been obtained in goats (Romeyer et al., 1994; Poindron et al., 2007). By contrast, the accessory olfactory system appears not to be involved in offspring recognition since the section of the accessory olfactory nerves does not induce deficits on the establishment of maternal selectivity in sheep (Lévy et al., 1995).

Ewes develop a selective bond with their newborn offspring, even when direct physical contact is prevented, providing that they have access to the lamb's salient odor (Poindron and Le Neindre, 1980; Romeyer et al., 1993; Otal et al., 2009), thus suggesting that lamb's olfactory signature is partly volatile. The recognizable odor profile of a lamb reflects a complex mosaic of chemical by-products of bodily processes. Indeed, a list of 133 volatile organic compounds associated with the wool of Döhne Merino lambs that are presumably involved in offspring recognition has been recently identified (Burger et al., 2011). Quantitative analysis and comparison of odor profiles reveal that the wool volatiles of twins are remarkably similar but that they differ from those of other twins or non-twin lambs. Unfortunately, when alien lambs are dressed in jackets sprayed with synthetic mixtures formulated to match the chemical composition of the scents of the ewes' own lambs, ewes reject these alien lambs (Burger et al., 2011).

The functional role of the MOB has been confirmed using various pharmacological, neurochemical, and electrophysiological approaches. Electrophysiological recordings from MOB mitral cells were first performed in awake ewes before and after birth (Kendrick et al., 1992). During pregnancy, none of the MOB mitral cells respond to lamb related odors (lamb, amniotic fluids…), but they respond to food odors. After birth, there is a noticeable increase in the number of mitral cells that responded to lamb odors, suggesting that the change in salience of the lamb odor that occurs at parturition is mediated by a shift in olfactory cell responsivity. Interestingly, among these MOB mitral cells, a small propotion responded preferentially to the odor of the familiar lamb, showing that already at the level of the MOB, a coding for the familiar lamb odor takes place.

Microdialysis analyses reveal that these changes in electrical properties of MOB mitral cells at parturition are the consequences of changes in the release of two neurotransmitters, the inhibitory gamma-amino-butyric-acid (GABA) and the excitatory glutamate within the MOB. Once ewes establish a selective bond with their lambs after parturition, the odors of familiar lambs, but not those of unfamiliar ones, increase the release of both transmitters. Infusion of the GABAa receptor antagonist bicuculline in the MOB prevents lamb recognition once it has been formed. Therefore, it is hypothesized that the general increase of GABA refines the olfactory signal by inhibiting MOB mitral cells with the exception of those processing the odor of the familiar lamb.

A dramatic increase of noradrenaline release also occurs during the learning of lamb odor (Lévy et al., 1993). Lesions of noradrenergic projections to the MOB or direct infusions of ß-adrenergic antagonist reduce the number of ewes developing the olfactory memory, without disrupting maternal care to the lamb (Pissonnier et al., 1985; Lévy et al., 1990). Contrary to the inhibitory effects of GABA on MOB mitral cell activity, the release of noradrenaline at birth induces the disinhibition of MOB mitral cells, thus allowing potentiation of the glutamatergic system by the retrograde messenger, nitric oxide (Kendrick et al., 1997).

In addition to these neurochemical changes, olfactory neurogenesis could provide another mechanism through which olfaction can contribute to lamb recognition. The olfactory bulb is a brain region where new neurons are continuously added during adult life. Production of new cells occurs at the level of the sub-ventricular zone (SVZ; Lois and Alvarez-Buylla, 1994; Doetsch et al., 1997, 1999) which contains neuronal stem cells that generate neuroblasts which then migrate through the rostral migratory stream toward the MOB. Once in the MOB, neuroblasts migrate radially in the different layers of the MOB, especially in the granular cell layer and to a lower extent in the periglomerular cell layer where these cells start to differentiate into mature GABAergic neurons expressing specific phenotypic markers such as NeuN (Lledo et al., 2006). During maturation, it is noticeable that around 50% of new cells die while the remaining half survives and is integrated into the olfactory network. This survival is highly regulated by both external and internal factors (Lledo and Saghatelyan, 2005). In sheep, as in rodents, neural stem cells proliferate on the margins of the SVZ and a rostral migratory stream is evidenced from the SVZ up to the MOB consisting of neuroblasts which form chain-like structures. The neuroblasts differentiate into mainly granular neurons once they have reached the MOB (Brus et al., 2010, 2012). Unlike the SVZ of rodents, the SVZ of sheep is particularly expanded to the open olfactory ventricle in the MOB (Luzzati et al., 2003; Brus et al., 2010), suggesting that the migration pathway of adult-born cells follows this ventricle up the MOB. In addition, the time required for maturation of adult-born cells in the sheep MOB is around four months, much longer than that of rodents (Brus et al., 2012).

The capacity of the olfactory system to generate new interneurons is thought to play an important function in social situations where olfaction plays a pivotal role (Gheusi et al., 2009; Lévy et al., 2011a). In the context of maternal behavior in sheep, regulation of neurogenesis has been reported at the time of parturition and according to mother-young interactions. Mothers having interactions with their lamb for 2 days exhibit less cell proliferation in the SVZ and fewer neuroblasts in the MOB in comparison to non-pregnant females (Brus et al., 2010; Lévy et al., 2011b). Although these results are correlational by nature, we hypothesize that this down-regulation of neurogenesis could facilitate learning of lamb odor by decreasing cell competition and favoring maturation of surviving new neurons. Future work should validate this hypothesis by using more functional approaches including for example the use of anti-mitotic drugs such as Arabinofuranosyl Cytidine (ara-C) and examine the behavioral consequences of the blockade of adult olfactory neurogenesis.

Downstream to the level of the MOB, the identification of activated brain regions during olfactory bonding confirms the importance of the main olfactory system. Indeed, a comparison of the c-fos mRNA expression in mothers exposed to a lamb after parturition to non-gestant females receiving an artificial vaginocervical stimulation (mimicking the expulsion of the fetus) without being in contact with a lamb has been performed. It showed an increase in Fos expression that was mainly restricted to the main olfactory processing regions, i.e., the MOB, the piriform cortex, the frontal medial cortex and the orbitofrontal cortex in females exposed to lambs (Da Costa et al., 1997). Similar results were obtained using Fos immunocytochemistry after exposing intact selective mothers to their lamb and comparing them to anosmic mothers showing no sign of individual lamb recognition (Keller et al., 2004a).

**Figure 1:**
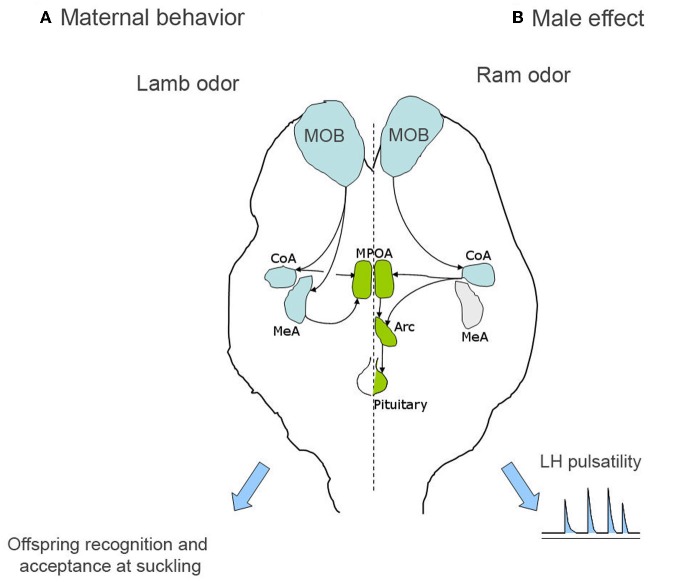
**Simplified representation of the processing of olfactory information in the case of both offspring recognition and male effect in sheep. (A)** In the case of offspring recognition, lamb odor is processed by the main olfactory bulb and then by both the medial and cortical nuclei of the amygdala, before to reach the medial preoptic area, which is involved in the expression of maternal care and offspring acceptance at suckling. **(B)** In the case of the male effect, the main olfactory bulb is also involved but then only the cortical amygdala seems to be in charge of the processing of male odor. The final output is the GnRH pulse generator at least partly localized in the arcuate nucleus which controls LH pulsatility and the reactivation of the gonadotrope axis. Arc, arcuate nucleus; CoA, cortical amygdala; MeA, medial amygdala; MOB, main olfactory bulb; MPOA, medial preoptic area.

Because the quantification of Fos expression is only a correlational method, the functional role of some brain structures showing Fos activation during the formation of olfactory lamb memory, were explored by using reversible pharmacological manipulation, revealing an important role for both the medial and the cortical nuclei of the amygdala (Keller et al., 2004b; Figure 1), which receive olfactory inputs from the MOB (Meurisse et al., 2009). Infusion of the anaesthetic lidocaïne, during the first 8 h post-partum, in either of these nuclei prevents animals from learning to discriminate their own lamb from an alien lamb and hence, both are permitted to suckle. This effect does not result from a disturbance of maternal acceptance or from an inability of memory retrieval.

Taken together, these results lead to the conclusion that the main olfactory system is involved in the control of lamb recognition at parturition. However, results showing that the AOB shows zif268 (an immediate early gene used as a marker of cellular activation) activation around parturition (Keller, 2003) and that the cauterization of the VNO entrance impairs maternal selectivity (Booth and Katz, 2000) raise the question of a possible involvement of the accessory olfactory system in offspring recognition. Because activation of AOB neurons requires a direct contact with the odorant source (Luo et al., 2003), and ewes exposed to olfactory cues but without any physical contact with the lamb develop a selective bond, the involvement of AOB in maternal selectivity is unlikely. The fact that the VNO can be activated by small volatile chemosignals (Leinders-Zufall et al., 2000; Trinh and Storm, 2003) prompts a more precise comparison of the effects of vomeronasal nerves section (Lévy et al., 1995) to those of cauterization of the vomeronasal duct (Booth and Katz, 2000). These authors also report that zinc sulfate infusion is ineffective in disrupting selectivity (Booth and Katz, 2000). However, regarding the main olfactory system, this discrepancy is probably due to methodological differences and to the small amounts of zinc sulfate solution used by Booth and Katz. Indeed, the application of around 2 mL of 1.5% ZnSO4 solution per nostril induces incomplete lesions of the main olfactory epithelium in many animals (in comparison with 50 mL of 1.5% ZnSO4 used by Poindron et al.), therefore failing to induce anosmia in an reliable way. This is, however, no longer the case when using a higher concentration of zinc sulfate (2.5%, Poindron et al., 2003).

## Olfactory systems involved in the male effect

In ungulates, the introduction of a male among seasonally anoestrous females results in activation of LH secretion (short-term response) leading later to ovulation and sexual receptivity (long-term response; Delgadillo et al., 2009). This phenomenon is commonly known as the “male effect” and is frequently used to advance and synchronize reproduction in sheep and goats. Contact with the male is not necessary to observe the reactivation of the gonadotropic axis since the short-term response can be fully mimicked by exposing the females to the male odor (Knight and Lynch, 1980).

The chemosignal responsible for the reactivation of the female gonadotropic axis seems to be a mixture of various compounds which have only been partially identified. It has been shown that the biological activity of the chemosignal requires the simultaneous presence of compound retained in both acid and neutral fractions (Cohen-Tannoudji et al., 1994). Recent work has focused on identification of the male odor in goats using changes in multiple unit activity as a “real-time” bioassay of neural activity in female goats (Hamada et al., 1996). This approach has yielded important perspectives on the nature of the male odor in this species: (1) odor production is dependent upon testosterone and is localized to the head, neck, and shoulders of the male (Hamada et al., 1996; Iwata et al., 2000; Wakabayashi et al., 2000); (2) the odorant activity resides in the lipid fraction of fleece extract (Okamura and Mori, 2005); (3) fleece odor from male sheep induces multiple unit electrophysiological activity and an associated increase in LH secretion in female goats (Ichimaru et al., 2008); and (4) 4 ethyloctanoic acid, the chemical responsible for the strong odor of male goats, elicit LH activity only when left for several months at room temperature (Iwata et al., 2003), suggesting a bacterial fermentation of precursors.

The role of the main and the accessory olfactory systems have been evaluated through lesioning or inactivating different regions of the main or the accessory olfactory pathways. Destruction of the main olfactory epithelium through intranasal administration of zinc sulfate or inactivation of the cortical nucleus of the amygdala by infusion of the anaesthetic lidocaine completely blocks the neuroendocrine response to ram odor (Gelez and Fabre-Nys, 2004, 2006b; Gelez et al., 2004; Figure 1). By contrast, lesion of the VNO or inactivation of the medial nucleus of the amygdala does not disrupt the response of females (Cohen-Tannoudji et al., 1989; Gelez and Fabre-Nys, 2006b).

Correlational studies using Fos immunocytochemistry to reveal central activations triggered by male odors support the view that the main olfactory system primarily conveys the male odor. In sheep, when comparing groups exposed to a male, male fleece, female fleece or no odor, the male or its odor significantly increases Fos expression in the main olfactory system, especially the MOB and the cortical nucleus of the amygdala (Gelez and Fabre-Nys, 2006a). However, the AOB, but no downstream structures, express Fos immunoreactivity following exposure to the ram odor. The AOB could play a minor role in the detection of the ram odor by activating the cortical nucleus of the amygdala through their reciprocal connections (Meurisse et al., 2009).

In goats, neuroendocrine activations by male odor involve the kisspeptin system in the arcuate nucleus of the hypothalamus (Hamada et al., 1996; Murata et al., 2009, 2011; Okamura et al., 2010). These kisspeptin cells are thought to be the intrinsic source of the GnRH pulse generator which consequently leads to LH release and ovulation. However, the olfactory system involved in the modulation of kisspeptin neurons has not been explored in the goat. The activation of the gonadotrope axis by male odor is confirmed by the fact that at the downstream stage, exposure to male odor is correlated with Fos activation of GnRH neurons in sheep (Gelez and Fabre-Nys, 2006a).

In summary, it has been shown that in sheep, contrary to many other species, the main olfactory system is primarily involved in the processing of the olfactory signal emanating from the male and that mediates a physiological response, while the accessory olfactory system seems to be less engaged. This is in contrast to rodents, where pheromonal cues are usually processed by the vomeronasal system (Keller et al., 2009). For example, sexual partner odor induces Fos activation in the AOB rather than the MOB in mice (Halem et al., 2001). In the female rat, removal of the VNO blocks the neuroendocrine response induced by male urine (Beltramino and Taleisnik, 1983). To the best of our knowledge, the few examples in which mammalian pheromonal signals are detected and processed by the main olfactory system relates to behavioral responses rather than physiological effects. This is the case for the rabbit mammary pheromone that induces the nipple search behavior (Hudson and Distel, 1983; Charra et al., 2012) and for the chemosignals contained in boar saliva that elicit receptivity posture in female pigs (Dorries et al., 1997).

## A role for vomeronasal olfaction in ungulates?

The data presented in the context of maternal behavior as well as those related to the male effect clearly support a key role for the main olfactory system in ungulate social behavior. Evidence showing an involvement of the vomeronasal system is scarce and the only experiment claiming a role of the VNO in sheep offspring recognition has raised methodological concerns.

However, ungulates are one of the animal taxa where a specific olfactory behavior that is thought to be dependent upon the vomeronasal system, namely flehmen response, is widely reported (Melese-d'Hospital and Hart, 1985). This behavior is highly expressed at the time of mother–young interactions at birth and during sexual encounters. Indeed, flehmen behavior is evoked most readily by olfactory investigation of urine, vaginal, or amniotic fluid secretions, and is believed to be involved in the transport of fluid-borne chemical stimuli, such as sexual odors, from the oral cavity to the VNO (Melese-d'Hospital and Hart, 1985).

At the neuroanatomical level, the VNO, the AOB, and the “vomeronasal” amygdala have been identified and are quite well developed in many wild and farm ungulate species (Kratzing, 1971; Salazar et al., 2007; Vedin et al., 2010). Both VNO and AOB complete their morphological development around the last third of the gestation period (Salazar et al., 2003), and a specific lectin for oligomeric N-acetylglucosamine labels the sensory epithelium of the VNO, the vomeronasal nerves, and the nervous and glomerular layers of the AOB before birth, thus suggesting that the vomeronasal system may be able to function at or even before birth (whereas in rodents this is precluded by the AOB not completing its development before birth).

As in other species, in sheep the chemosignals are thought to contact vomeronasal receptors in the VNO epithelium through a pumping mechanism (Meredith et al., 1980; Melese-d'Hospital and Hart, 1985). The morphological features of the vomeronasal arteries and veins together with the existence of large autonomic and sensory innervations suggest that these vessels function similarly to erectile tissue (Salazar et al., 1998). The VNO sensory epithelium of sheep was studied at the morphological level using both optical and electronic microscopy (Kratzing, 1971) and showed only slight differences to that of rodents. However, if the vomeronasal system of ungulates seems to be perfectly functional, it is striking to notice that at the molecular level its importance seems to be quite reduced. Indeed, the ratio of the volume of the vomeronasal epithelium to that of the whole VNO in the goat is 8% in comparison to that found in the mouse (Wakabayashi et al., 2002). The size of goat V1R gene family is also smaller than that of rodent V1R gene families (Wakabayashi et al., 2002). In addition, most goat V2R gene products may not function as receptors since the V2R genes have multiple termination codons within their coding sequences (Wakabayashi et al., 2002). As a whole, the possibility exists that the VNO has evolved so that it has lost part of its role in the detection of social odors in ungulates and therefore the main olfactory system contributes mainly to the discrimination of these odors. Interestingly, the expression of one gene receptor (gV1ra1) of the V1R receptor family, a family of genes which is usually expressed in the VNO of rodents, has been found to be expressed in the main olfactory epithelium in goat (Wakabayashi et al., 2002, 2007).

The downstream organization of the vomeronasal system also seems to be perfectly functional, even if some slight differences with rodents can be noticed. First, the respective zone to zone projection from the apical and basal sensory epithelium of the VNO to the anterior and posterior part of the AOB, typical in rodents, is not present in adult sheep (Salazar et al., 2007) and goats (Takigami et al., 2000). With regard to the sheep AOB, its size seems to be relatively small in comparison to the MOB (Mesiami and Bhatnagar, 1998), and one of the AOB most prominent anatomical features is the scarce population of mitral/tufted cells and their dispersion (Salazar et al., 2007). A morphological consequence is that there is no clear presence of a classical plexiform layer in the stratification of the sheep AOB.

Finally, at the level of the central projections of the vomeronasal system, it seems that the vomeronasal amygdala of the sheep is as extensive as that of rodents (Jansen et al., 1998; Lévy et al., 1999) and the existence of vomeronasal projections to the medial amygdala has been documented (Jansen et al., 1998; Meurisse et al., 2009) and suggest a role of the vomeronasal system in ungulate social behavior.

In conclusion, the current knowledge on the regulation of social behavior by the accessory olfactory system leads to an apparent contradiction. Indeed, neuroanatomical characterizations suggest that despite a reduced relative size, the vomeronasal system seems to be perfectly functional. By contrast, the behavioral evidence regarding its function in social olfaction is scarce, therefore advocating for further investigations in this area. Particularly, the use of other behavioral situations than the ones explored so far could lead to a re-evaluation of the role of the vomeronasal olfaction in the control of social behavior in ungulates. For example, it is likely that vomeronasal olfaction could play a more developed role in wild ungulates such as antelopes or moose than in domesticated species (Deutsch and Nefdt, 1992; Vedin et al., 2010).

### Conflict of interest statement

The authors declare that the research was conducted in the absence of any commercial or financial relationships that could be construed as a potential conflict of interest.
